# Dose of Inhaled Corticosteroid in Chronic Obstructive Pulmonary Disease and Risks of Osteoporosis or Fracture—A Systematic Review and Meta‐Analysis

**DOI:** 10.1111/crj.70086

**Published:** 2025-05-26

**Authors:** Wang Chun Kwok, Chung Ki Tsui, Sze Him Isaac Leung, Shuk Man Ngai, David Chi Leung Lam, Mary Sau Man Ip, James Chung Man Ho

**Affiliations:** ^1^ Department of Medicine The University of Hong Kong, Queen Mary Hospital Hong Kong Special Administrative Region China; ^2^ Department of Statistics The Chinese University of Hong Kong Hong Kong Special Administrative Region China

**Keywords:** bone mineral density, COPD, fracture, inhaled corticosteroid, osteoporosis

## Abstract

**Background:**

Inhaled corticosteroid (ICS) is a major pharmacotherapy for chronic obstructive pulmonary disease (COPD), which is associated with various adverse effects. Controversies exist in whether ICS use in COPD is associated with osteoporosis or fracture.

**Objective:**

We performed a systematic review and meta‐analysis to assess the risks of osteoporosis or fracture at different dosing levels of ICS. High‐, medium‐ and low‐dose ICS were defined according to the Global Initiative for Asthma (GINA) step definition.

**Data sources:**

Cochrane, EMBASE, Ovid, PubMed and Web of Science were systematically searched until 8 December 2023.

**Data extraction:**

Osteoporosis or fracture under ICS therapy was chosen as the primary efficacy outcome. Three reviewers were involved independently in the extraction process. The risk of bias of the included studies was evaluated by using different assessment tools.

**Results:**

Twenty‐one RCTs and eight observational studies were included. High‐dose ICS was associated with increased risks of osteoporosis or fracture in RCTs with RR of 1.14 (95% CI = 1.03–1.28), observational studies with healthy controls 1.14 (95% CI = 1.05–1.24) and observational studies without healthy controls 1.10 (95% CI = 1.01–1.21). High‐dose ICS was associated with increased risks in fracture in RCTs with RR of 1.12 (95% CI = 1.03–1.23), observational studies with health controls 1.15 (95% CI = 1.05–1.25) and observational studies without healthy controls 1.13 (95% CI = 1.03–1.24). Medium‐ and low‐dose ICS were not associated with osteoporosis or fracture.

**Conclusion:**

High‐dose, but not medium‐ and low‐dose, ICS use in COPD is associated with risks of osteoporosis or fractures.

## Graphical abstract

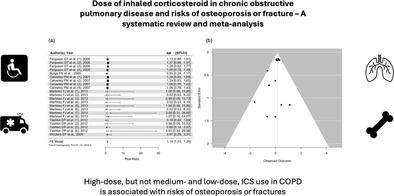

## 1 Introduction

Inhaled corticosteroid (ICS) has been one of the major pharmacotherapies for chronic obstructive pulmonary disease (COPD), providing clinical benefits by slowing down the decline in lung function [1, 2, 3, 4, 5], symptomatic improvement [2, 3, 6] and exacerbation reduction [2, 3, 6, 7, 8, 9]. However, the adverse effects associated with ICS use should not be overlooked. The commonly reported adverse effects from ICS include adrenal suppression [10], pneumonia [11], tuberculosis [12], non–tuberculosis mycobacterial infection [13] and cataract [14].

Osteoporosis is an important comorbidity in COPD [15]. Given the well‐reported association between systemic corticosteroid use and risks of osteoporosis or fracture [16], there was a concern on the same adverse effects with ICS use. However, the evidence supporting this remains controversial. A recently published systemic review and meta‐analysis revealed that ICS use was not shown to be associated with increased incidence of fracture or osteoporosis in subjects with COPD [17]. Another systemic review and meta‐analysis showed the opposite result [18]. On the other hand, systemic review and meta‐analysis suggested a dose–response relationship for the risk of non–vertebral fracture with ICS [19].

In view of these controversial results, it is crucial to examine this important clinical question again, by analysing the data with the focus on dose–response relationship.

## 2 Study Design and Methods

The meta‐analysis was conducted according to the Quality of Reporting of Meta‐Analyses guidelines [20].

### 2.1 Data Sources and Search Strategy

We searched Cochrane, EMBASE, Ovid, PubMed and Web of Science until 8 December 2023, to identify potentially relevant observational studies and RCTs. Our search included combined medical subject headings and keywords for adults with COPD (study population), use of an ICS (study intervention or exposure of interest) and osteoporosis or fracture of any bone. Additionally, manual search of reference lists of the selected articles was performed to identify additional studies and to ensure comprehensive search.

### 2.2 Study Selection

Two reviewers (W. C. K. and C. K. T., who are the authors of this systemic review and meta‐analysis) independently screened studies for inclusion, retrieved potentially relevant studies and determined study eligibility. Disagreements were resolved by group consensus. Included observational studies and RCTs were those having enrolled adults with clinically diagnosed COPD and outcomes of interest being reported cases of fracture, osteoporosis and/or decreased BMD. Individuals without ICS exposure were also included as control subjects. Studies enrolling patients mixed with other respiratory diseases except COPD and without an ICS unexposed comparison group were excluded. All studies included in the meta‐analysis contained an ICS exposed and an ICS unexposed group for comparison. Patients who had been exposed to ICS, even if they were receiving mixed therapy with long‐acting beta agonists (LABA) and/or long‐acting muscarinic antagonists (LAMA) at the same time, were classified into the ICS‐exposed group. Patients who were not exposed to ICS were either receiving no drug prescription or treated with LABA and/or LAMA. Agreement between reviewers regarding study inclusion was assessed using the Cohen κ statistic [21].

### 2.3 Data Extraction

Two reviewers (S. H. L. and C. K. T.) independently abstracted data and methods from included studies using custom‐made standardized forms. Extracted data were entered into Microsoft Excel (Microsoft Corp, Redmond, Washington) and were checked by a third reviewer (W. C. K.). Abstracted data included study design (e.g., date of conduct and sample size), patient characteristics and study methodology (e.g., eligibility criteria, method of randomization and blinding), intervention (e.g., ICS type, dose and duration; outcome definitions) and main results. To compare the effects of different doses of ICS, the ICS doses in each study were classified according to the GINA step definition. The studies that compared different types or doses of ICS with various control groups were split into subgroups to evaluate the effect of ICS dosage for each type of exposed and unexposed control groups. Disagreements between the two interviewers were resolved by group consensus. Methodological quality of included studies was evaluated by collecting data on sources of systematic bias according to the published guidelines [22]. These data included the description of sequence generation, allocation concealment, assessor blinding, incomplete outcome data, selective reporting, eligibility criteria, therapies and excluded patients. We also quantified study quality using the Jadad score [23], Cochrane bias assessment method [24] and National Institutes of Health Quality Assessment Tool [25].

The primary outcome of this meta‐analysis was the development of osteoporosis or fracture after high‐, medium‐ and low‐dose ICS therapy.

### 2.4 Statistical Analysis

Dichotomous outcomes were reported as relative risks (RRs) and the respective 95% confidence intervals (CI). The *I* ^*2*^ statistic was calculated to determine the proportion of between‐study variation due to heterogeneity, with suggested thresholds for low (25%–49%), moderate (50%–74%) and high (≥ 75%) values [26]. In analyses with low heterogeneity(*I* ^*2*^ ≤ 50%), a fixed‐effects model was used, while random‐effects model was used for high heterogeneity analysis (*I* ^*2*^ ≥ 50%). Publication bias was assessed using funnel plots and the Egger's test. All statistical analyses were performed using R Studio software (2022.07.2 + 576).

## 3 Results

**FIGURE 1:**
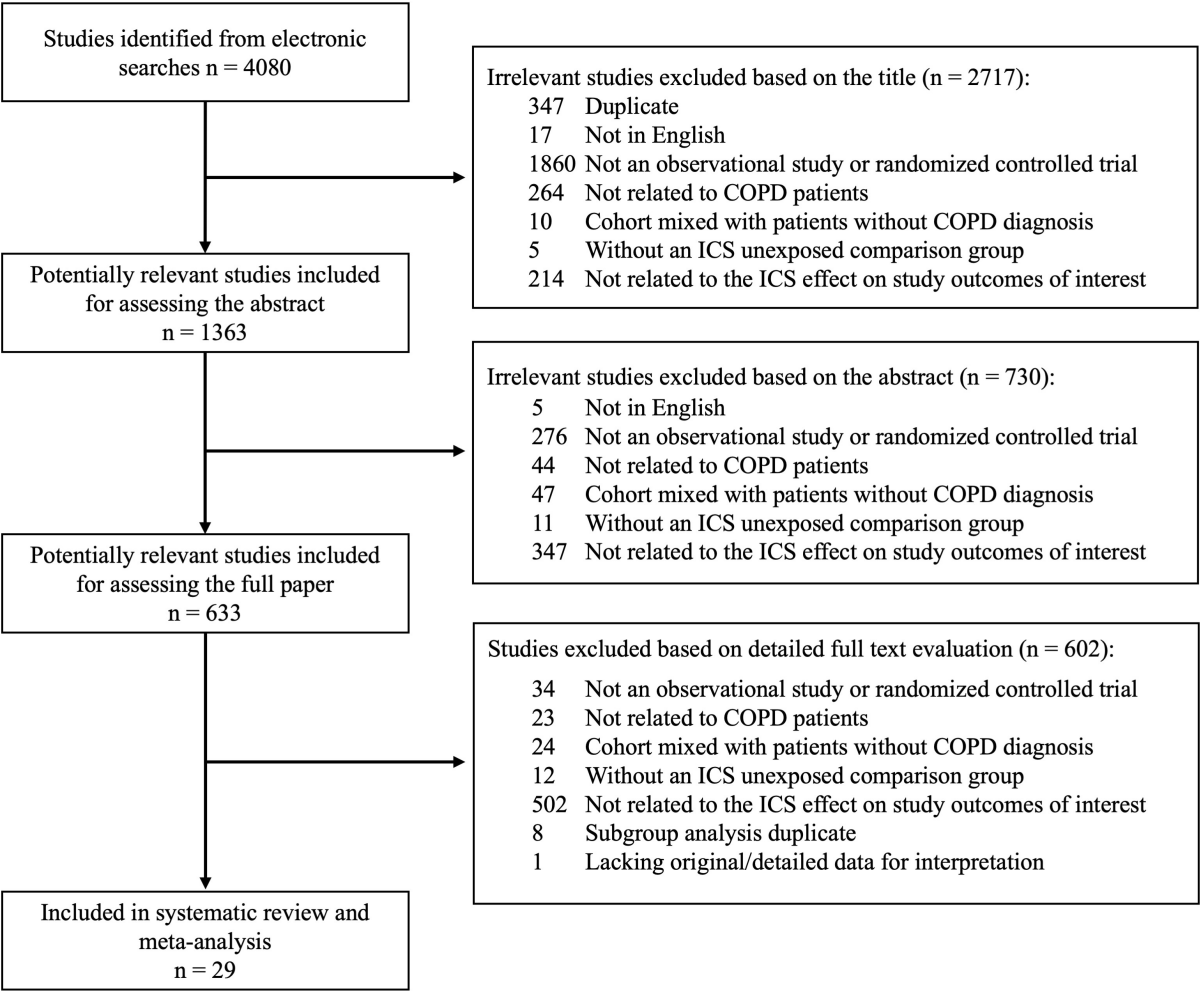
Flowchart of the selection process.

The electronic database searches identified 4080 citations. After evaluating these citations, review articles and the bibliographies of included studies, we included 21 randomized controlled trials [1, 2, 3, 7, 8, 27, 28, 29, 30, 31, 32, 33, 34, 35, 36, 37, 38, 39, 40, 41, 42] and eight observational studies [43, 44, 45, 46, 47, 48, 49, 50] (Figure 1). Among the RCTs, five reported osteoporosis or fracture as a primary outcome [27, 30, 32, 33, 36], and 16 reported osteoporosis or fracture as a secondary outcome [1, 2, 3, 7, 8, 28, 29, 31, 34, 35, 37, 38, 39, 40, 41, 42]. The Cohen κ statistic for agreement on study inclusion was 0.91. We conducted separate meta‐analyses among observational studies (with different dosages, fractures and osteoporosis) and RCTs (with different dosages, fractures and osteoporosis) to obtain the pooled RRs. The characteristics of the included studies are summarized in Tables 1 and 2.

**TABLE 1:** Characteristics of the included RCTs in forest plots.

Author(s), Year	COPD severity	Outcome	ICS‐exposed treatment drug	ICS‐unexposed treatment	Dosage used in trials
RCTs					
Ferguson GT et al. (1), 2009 [27]	Moderate to severe	Fractures	Fluticasone propionate	Placebo	High
Ferguson GT et al. (2), 2009 [27]		Fractures	Salmeterol/fluticasone propionate	Placebo	High
Ferguson GT et al. (3), 2009 [27]		Fractures	Salmeterol/Fluticasone propionate	Salmeterol	High
Ferguson GT et al. (4), 2009 [27]		Fractures	Fluticasone propionate	Salmeterol	High
Pepin JL et al., 2014 [28]	Moderate to severe	Fractures	Fluticasone furoate/vilanterol	Tiotropium bromide	Low
Tashkin DP et al. (1), 2008 [29]	Moderate to severe	Fractures	Budesonide/formoterol 160/4.5 μg	Formoterol 4.5 μg	Low
Tashkin DP et al. (2), 2008 [29]		Fractures	Budesonide/formoterol 160/4.5 μg	Placebo	Low
Tashkin DP et al. (3), 2008 [29]		Fractures	Budesonide/formoterol 80/4.5 μg	Formoterol 4.5 μg	Low
Tashkin DP et al. (4), 2008 [29]		Fractures	Budesonide/formoterol 80/4.5 μg	Placebo	Low
Tashkin DP et al. (5), 2008 [29]		Fractures	Budesonide 160 μg + formoterol 4.5 μg	Formoterol 4.5 μg	Low
Tashkin DP et al. (6), 2008 [29]		Fractures	Budesonide 160 μg + formoterol 4.5 μg	Placebo	Low
Tashkin DP et al. (7), 2008 [29]		Fractures	Budesonide 160 μg	Formoterol 4.5 μg	Low
Tashkin DP et al. (8), 2008 [29]		Fractures	Budesonide 160 μg	Placebo	Low
Maltais F et al., 2020 [30]	Moderate	Fractures	Fluticasone furoate/vilanterol 100 μg/25 μg	Vilanterol 25 μg/day	Low
Rabe KF et al. (1), 2020 [7]	Moderate to severe	Fractures	Budesonide/glycopyrrolate/formoterol 320/18/9.6 μg	Glycopyrrolate/formoterol 18/9.6 μg	Medium
Rabe KF et al. (2), 2020 [7]		Fractures	Budesonide/flycopyrrolate/formoterol 160/18/9.6 μg	Glycopyrrolate/formoterol 18/9.6 μg	Low
Rabe KF et al. (3), 2020 [7]		Fractures	Budesonide/formoterol 320/9.6 μg	Glycopyrrolate/formoterol 18/9.6 μg	Medium
Burge PS et al., 2000 [2]	Moderate to severe	Fractures	Fluticasone propionate 500 ug	Placebo	High
Calverley PM et al. (1), 2007 [3]	Moderate to severe	Fractures	Salmeterol 50 μg/fluticasone propionate 500 μg	Salmeterol	High
Calverley PM et al. (2), 2007 [3]		Fractures	Salmeterol 50 μg/fluticasone propionate 500 μg	Placebo	High
Calverley PM et al. (3), 2007 [3]		Fractures	Fluticasone propionate	Salmeterol	High
Calverley PM et al. (4), 2007 [3]		Fractures	Fluticasone propionate	Placebo	High
Ferguson GT et al. (1), 2017 [31]	Moderate to severe	Fractures	Budesonide/formoterol 320/9 mg	Formoterol 9 mg	Medium
Ferguson GT et al. (2), 2017 [31]		Osteoporosis	Budesonide/formoterol 320/9 mg	Formoterol 9 mg	Medium
Johnell O et al., 2002 [32]	Mild	Fractures	Budesonide 400 mg	Placebo	Medium
Kerwin EM et al. (1), 2019 [33]	Moderate to severe	Osteoporosis	Budesonide/glycopyrrolate/formoterol fumarate 320/18/9.6 μg	Glycopyrrolate/formoterol fumarate 18/9.6 μg	Medium
Kerwin EM et al. (2), 2019 [33]		Osteoporosis	Budesonide/formoterol fumarate 320/9.6 μg	Glycopyrrolate/formoterol fumarate 18/9.6 μg	Medium
Kerwin EM et al. (1), 2013 [34]	Moderate to severe	Fractures	Fluticasone furoate/vilanterol 100/25 mg	Vilanterol 25 mg	Low
Kerwin EM et al. (2), 2013 [34]		Fractures	Fluticasone furoate/vilanterol 100/25 mg	Placebo	Low
Kerwin EM et al. (3), 2013 [34]		Fractures	Fluticasone furoate/vilanterol 50/25 mg	Vilanterol 25 mg	Low
Kerwin EM et al. (4), 2013 [34]		Fractures	Fluticasone furoate/vilanterol 50/25 mg	Placebo	Low
Kerwin EM et al. (5), 2013 [34]		Fractures	Fluticasone furoate 100 mg	Vilanterol 25 mg	Low
Kerwin EM et al. (6), 2013 [34]		Fractures	Fluticasone furoate 100 mg	Placebo	Low
Martinez FJ et al. (1), 2013 [35]	Moderate to severe	Fractures	Fluticasone furoate/vilanterol 100/25 ug	Vilanterol 25 mg	High
Martinez FJ et al. (2), 2013 [35]		Fractures	Fluticasone furoate/vilanterol 100/25 ug	Placebo	High
Martinez FJ et al. (3), 2013 [35]		Fractures	Fluticasone furoate/vilanterol 200/25 ug	Vilanterol 25 mg	High
Martinez FJ et al. (4), 2013 [35]		Fractures	Fluticasone furoate/vilanterol 200/25 ug	Placebo	High
Martinez FJ et al. (5), 2013 [35]		Fractures	Fluticasone furoate 100 ug	Vilanterol 25 mg	High
Martinez FJ et al. (6), 2013 [35]		Fractures	Fluticasone furoate 100 ug	Placebo	High
Martinez FJ et al. (7), 2013 [35]		Fractures	Fluticasone furoate 200 ug	Vilanterol 25 mg	High
Martinez FJ et al. (8), 2013 [35]		Fractures	Fluticasone furoate 200 ug	Placebo	High
Pauwels RA et al., 1999 [1]	Mild	Fractures	Budesonide 400ug	Placebo	Medium
Scanlon PD et al. (1), 2004 [36]	Mild to severe	Fractures	Triamcinolone acetonide 600 mcg	Placebo	Medium
Scanlon PD et al. (2), 2004 [36]		Osteoporosis	Triamcinolone acetonide 600 mcg	Placebo	Medium
Tashkin DP et al. (1), 2012 [37]	Moderate to severe	Fractures	Mometasone furoate/formoterol fumarate 400/10 μg	Formoterol fumarate 10 μg	High
Tashkin DP et al. (2), 2012 [37]		Fractures	Mometasone furoate/formoterol fumarate 400/10 μg	Placebo	High
Tashkin DP et al. (3), 2012 [37]		Fractures	Mometasone furoate/formoterol fumarate 200/10 μg	Formoterol fumarate 10 μg	Medium
Tashkin DP et al. (4), 2012 [37]		Fractures	Mometasone furoate/formoterol fumarate 200/10 μg	Placebo	Medium
Tashkin DP et al. (5), 2012 [37]		Fractures	Mometasone furoate/formoterol fumarate 400 μg	Formoterol fumarate 10 μg	High
Tashkin DP et al. (6), 2012 [37]		Fractures	Mometasone furoate/formoterol fumarate 400 μg	Placebo	High
Wang et al. (1), 2020 [42]	Moderate to severe	Osteoporosis	Budesonide/glycopyrrolate/formoterol 320/18/9.6 lg	Glycopyrrolate/formoterol fumarate 18/9.6 lg	Medium
Wang et al. (2), 2020 [42]		Osteoporosis	Budesonide/formoterol fumarate 320/9.6 lg	Glycopyrrolate/formoterol fumarate 18/9.6 lg	Medium
Wang et al. (3), 2020 [42]		Osteoporosis	Budesonide/formoterol fumarate 400/12 lg	Glycopyrrolate/gormoterol fumarate 18/9.6 lg	Medium
Wouters EF et al., 2005 [38]	Moderate to severe	Fractures	Salmeterol 50 ug/fluticasone 500 ug	Salmeterol 50ug	High
Bansal S et al., 2021 [39]	Moderate to severe	Fractures	Fluticasone furoate/umeclidinium/vilanterol 100/62.5/25 mcg	Tiotropium 18mcg	Low
Lipson DA et al. (1), 2018 [8]	Moderate to severe	Fractures	Fluticasone furoate/umeclidinium/vilanterol 100/62.5/25 μg	Umeclidinium– vilanterol 62.5/25 μg	Low
Lipson DA et al. (2), 2018 [8]		Fractures	Fluticasone furoate– vilanterol 100/25 μg	Umeclidinium– vilanterol 62.5/25 μg	Low
Bhatt SP et al. (1), 2017 [40]	Moderate to severe	Fractures	Fluticasone furoate 100/25 μg/vilanterol 25 μg	Placebo	Low
Bhatt SP et al. (2), 2017 [40]		Fractures	Fluticasone furoate 100/25 μg/vilanterol 25 μg	Vilanterol 25 μg	Low
Siler TM et al., 2017 [41]	Moderate to severe	Fractures	Fluticasone furoate/vilanterol 100/25 mg	Vilanterol 25 μg	Low

**TABLE 2:** Characteristics of the included observational studies in forest plots.

Observational studies					
Pujades‐Rodriguez M et al. (1), 2007 [43]	Mild to severe	Fractures	Mixed	/	Low
Pujades‐Rodriguez M et al. (2), 2007 [43]		Fractures	Mixed	/	Low
Pujades‐Rodriguez M et al. (3), 2007 [43]		Fractures	Mixed	/	Medium
Pujades‐Rodriguez M et al. (4), 2007 [43]		Fractures	Mixed	/	Medium
Pujades‐Rodriguez M et al. (5), 2007 [43]		Fractures	Mixed	/	High
Pujades‐Rodriguez M et al. (6), 2007 [43]		Fractures	Mixed	/	High
Lee TA et al. (1), 2004 [44]	Mild to severe	Fractures	Beclomethasone equivalents	/	Low
Lee TA et al. (2), 2004 [44]		Fractures	Beclomethasone equivalents	/	Medium
Lee TA et al. (3), 2004 [44]		Fractures	Beclomethasone equivalents	/	High
Suissa S et al. (1), 2004 [45]	Mild to severe	Fractures	Mixed	/	Low
Suissa S et al. (2), 2004 [45]		Fractures	Mixed	/	Medium
Suissa S et al. (3), 2004 [45]		Fractures	Mixed	/	High
Suissa S et al. (4), 2004 [45]		Fractures	Mixed	/	High
Suissa S et al. (5), 2004 [45]		Fractures	Mixed	/	High
Gonzalez AV et al. (1), 2018 [49]	Mild to severe	Fractures	Fluticasone equivalents	/	Medium
Gonzalez AV et al. (2), 2018 [49]		Fractures	Fluticasone equivalents	/	High
Gonzalez AV et al. (3), 2018 [49]		Fractures	Fluticasone equivalents	/	High
Chiu KL et al. (1), 2021 [46]	Mild to severe	Osteoporosis	Mixed	/	Low
Chiu KL et al. (2), 2021 [46]		Osteoporosis	Mixed	/	Medium
Chiu KL et al. (3), 2021 [46]		Osteoporosis	Mixed	/	High
Liu SF et al. (1), 2016 [47]	Mild to severe	Osteoporosis	Fluticasone propionate and/or budesonide	/	Low
Liu SF et al. (2), 2016 [47]		Osteoporosis	Fluticasone propionate and/or budesonide	/	Medium
Liu SF et al. (3), 2016 [47]		Osteoporosis	Fluticasone propionate and/or budesonide	/	High
Price DB et al. (1), 2019 [48]	Mild to severe	Osteoporosis	Mixed	/	Low
Price DB et al. (2), 2019 [48]		Osteoporosis	Mixed	/	Medium
Price DB et al. (3), 2019 [48]		Osteoporosis	Mixed	/	High
Janson C et al. (1), 2021 [50]	Mild to severe	Fractures	Budesonide or equivalent	/	Low
Janson C et al. (2), 2021 [50]		Osteoporosis	Budesonide or equivalent	/	Low
Janson C et al. (3), 2021 [50]		Fractures	Budesonide or equivalent	/	High
Janson C et al. (4), 2021 [50]		Osteoporosis	Budesonide or equivalent	/ (Healthy control)	High
Janson C et al. (5), 2021 [50]		Fractures	Budesonide or equivalent	/ (Healthy control)	Low
Janson C et al. (6), 2021 [50]		Fractures	Budesonide or equivalent	/ (Healthy control)	High
Janson C et al. (7), 2021 [50]		Osteoporosis	Budesonide or equivalent	/ (Healthy control)	Low
Janson C et al. (8), 2021 [50]		Osteoporosis	Budesonide or equivalent	/ (Healthy control)	High

### 3.1 Quality Assessment

In general, methodological quality of included RCTs was good. All included studies were assessed by the Cochrane bias assessment method and had a Jadad score of ≥ 3 (Table 3). All studies had clearly defined eligibility criteria, intervention details and reasons for patient exclusion. Allocation sequence generation and concealment were adequately described in 14 and 16 RCTs, respectively [2, 3, 7, 8, 27, 29, 30, 31, 33, 34, 35, 37, 38, 39, 40, 41, 42]. All studies demonstrated an adequate description of assessor blinding and reported statistical methods including sample‐size calculations. In total, 15 studies reported intention‐to‐treat analysis [1, 3, 7, 8, 28, 29, 31, 33, 34, 35, 38, 39, 40, 41, 42]. The risk of bias among the included observational studies was assessed by the National Institutes of Health Quality Assessment Tool and reported (Tables 4 and 5). Publication bias was assessed by inspection of the funnel plots as well as analysis of the Egger's regression test for each pooled analysis. There was no evidence of significant publication bias for the primary or secondary outcomes.

**TABLE 3:** Risk of bias assessment for included trials.^a^

Study	Random sequence generation	Allocation concealment	Blinding of participants and personnel	Blinding of outcome assessment	Incomplete outcome data addressed	Selective reporting	Other sources of bias	Jadad score^b^
Ferguson GT et al., 2009 [27]	Low	Low	Low	Low	Low	Low	Low	5
Pepin JL et al., 2014 [28]	Unclear	Unclear	Low	Unclear	Low	Low	Low	4
Tashkin DP et al., 2008 [29]	Low	Low	Low	Low	Low	Low	Low	5
Maltais F et al., 2020 [30]	Unclear	Low	Low	Unclear	Low	Low	Unclear	4
Rabe KF et al., 2020 [7]	Low	Unclear	Low	Low	Low	Low	Low	4
Burge PS et al., 2000 [2]	Low	Low	Low	Low	Low	Low	High	4
Calverley PM et al., 2007 [3]	Low	Low	Low	Low	Low	Low	Low	5
Ferguson GT et al., 2017 [31]	Low	Low	Low	Low	Low	Low	Low	5
Johnell O et al., 2002 [32]	Unclear	Unclear	Low	Unclear	Low	Low	Low	3
Kerwin EM et al., 2019 [33]	Low	Low	Low	Low	Low	Low	Low	5
Kerwin EM et al., 2013 [34]	Low	Low	Low	Low	Low	Low	Low	5
Martinez FJ et al., 2013 [35]	Low	Low	Low	Low	Low	Low	Low	5
Pauwels RA et al., 1999 [1]	Unclear	Unclear	Low	Low	Low	Low	Low	3
Scanlon PD et al., 2004 [36]	Unclear	Unclear	Low	Low	Low	Low	Low	3
Tashkin DP et al., 2012 [37]	Low	Low	Low	Low	Low	Low	Low	5
Wang C et al., 2020 [42]	Low	Low	Low	Low	Low	Low	Low	5
Wouters EF et al., 2005 [38]	Unclear	Low	Low	Low	Low	Low	Low	4
Bansal S et al., 2021 [39]	Low	Low	Low	Low	Low	Low	Low	5
Lipson DA et al., 2018 [8]	Unclear	Low	Low	Low	Low	Low	Low	4
Bhatt SP et al., 2017 [40]	Low	Low	Low	Low	Low	Low	Low	5
Siler TM et al., 2017 [41]	Low	Low	Low	Low	Low	Low	Low	5

^a^ Qualitative assessment was conducted using the Jadad score [23] and Cochrane bias assessment method [24]. Assessment of each criterion was ‘high risk of bias’, ‘low risk of bias’ or ‘unclear risk of bias’.

^b^ Range 1–5; 5 indicates highest quality [23].

**TABLE 4:** Risk of bias assessment for observational studies (for observational cohort and cross–sectional studies).^a^

Study	Q1	Q2	Q3	Q4	Q5	Q6	Q7	Q8	Q9	Q10	Q11	Q12	Q13	Q14
Liu SF et al., 2016 [47]	Yes	Yes	Yes	Yes	No	Yes	Yes	Yes	Yes	No	Yes	NA	Yes	Yes
Price DB et al., 2019 [48]	Yes	Yes	Yes	Yes	NR	Yes	Yes	Yes	Yes	No	Yes	NA	Yes	Yes
Janson C et al., 2021 [50]	Yes	Yes	Yes	Yes	Yes	Yes	Yes	Yes	Yes	No	Yes	NA	Yes	Yes

^a^ Qualitative assessment was conducted using the National Institutes of Health Quality Assessment Tool [25]. Assessment of each criterion was ‘Yes’, ‘No’, ‘CD: cannot determine’, ‘NA: not applicable’ or ‘NR: not reported’ [25].

Q1. Was the research question or objective in this paper clearly stated? Q2. Was the study population clearly specified and defined? Q3. Was the participation rate of eligible persons at least 50%? Q4. Were all the subjects selected or recruited from the same or similar populations (including the same time period)? Were inclusion and exclusion criteria for being in this study prespecified and applied uniformly to all participants? Q5. Were sample size justification, power description or variance and effect estimates provided? Q6. For the analyses in this paper, were the exposure(s) of interest measured prior to the outcome(s) being measured? Q7. Was the timeframe sufficient so that one could reasonably expect to see an association between exposure and outcome if it existed? Q8. For exposures that can vary in amount or level, did this study examine different levels of the exposure as related to the outcome (e.g., categories of exposure or exposure measured as continuous variable)? Q9. Were the exposure measures (independent variables) clearly defined, valid, reliable and implemented consistently across all study participants? Q10. Was the exposure(s) assessed more than once over time? Q11. Were the outcome measures (dependent variables) clearly defined, valid, reliable and implemented consistently across all study participants? Q12. Were the outcome assessors blinded to the exposure status of participants? Q13. Was loss to follow‐up after baseline 20% or less? Q14. Were key potential confounding variables measured and adjusted statistically for their impact on the relationship between exposure(s) and outcome(s)?

**TABLE 5:** Risk of bias assessment for observational studies (for case–control studies).^a^

Study	Q1	Q2	Q3	Q4	Q5	Q6	Q7	Q8	Q9	Q10	Q11	Q12
Pujades‐Rodriguez M et al., 2007 [43]	Yes	Yes	No	Yes	Yes	Yes	NA	Yes	Yes	Yes	Yes	Yes
Lee TA et al., 2004 [44]	Yes	Yes	No	Yes	Yes	Yes	NA	Yes	Yes	Yes	Yes	Yes
Suissa S et al., 2004 [45]	Yes	Yes	No	Yes	Yes	Yes	NA	Yes	Yes	Yes	Yes	Yes
Gonzalez AV et al., 2018 [49]	Yes	Yes	No	Yes	Yes	Yes	NA	Yes	Yes	Yes	Yes	Yes
Chiu KL et al., 2021 [46]	Yes	Yes	No	Yes	Yes	Yes	NA	Yes	Yes	Yes	Yes	Yes

^a^ Qualitative assessment was conducted using the National Institutes of Health Quality Assessment Tool [25]. Assessment of each criterion was ‘Yes’ ‘No’, ‘CD: cannot determine’, ‘NA: not applicable’ or ‘NR: not reported’ [25].

Q1. Was the research question or objective in this paper clearly stated and appropriate? Q2. Was the study population clearly specified and defined? Q3. Did the authors include a sample size justification? Q4. Were controls selected or recruited from the same or similar population that gave rise to the cases (including the same timeframe)? Q5. Were the definitions, inclusion and exclusion criteria, algorithms or processes used to identify or select cases and controls valid, reliable and implemented consistently across all study participants?

Q6. Were the cases clearly defined and differentiated from controls? Q7. If less than 100% of eligible cases and/or controls were selected for this study, were the cases and/or controls randomly selected from those eligible? Q8. Was there use of concurrent controls? Q9. Were the investigators able to confirm that the exposure/risk occurred prior to the development of the condition or event that defined a participant as a case? Q10. Were the measures of exposure/risk clearly defined, valid, reliable and implemented consistently (including the same time period) across all study participants? Q11. Were the assessors of exposure/risk blinded to the case or control status of participants? Q12. Were key potential confounding variables measured and adjusted statistically in the analyses? If matching was used, did the investigators account for matching during the study analysis?

### 3.2 From Randomized Controlled Trials

**FIGURE 2:**
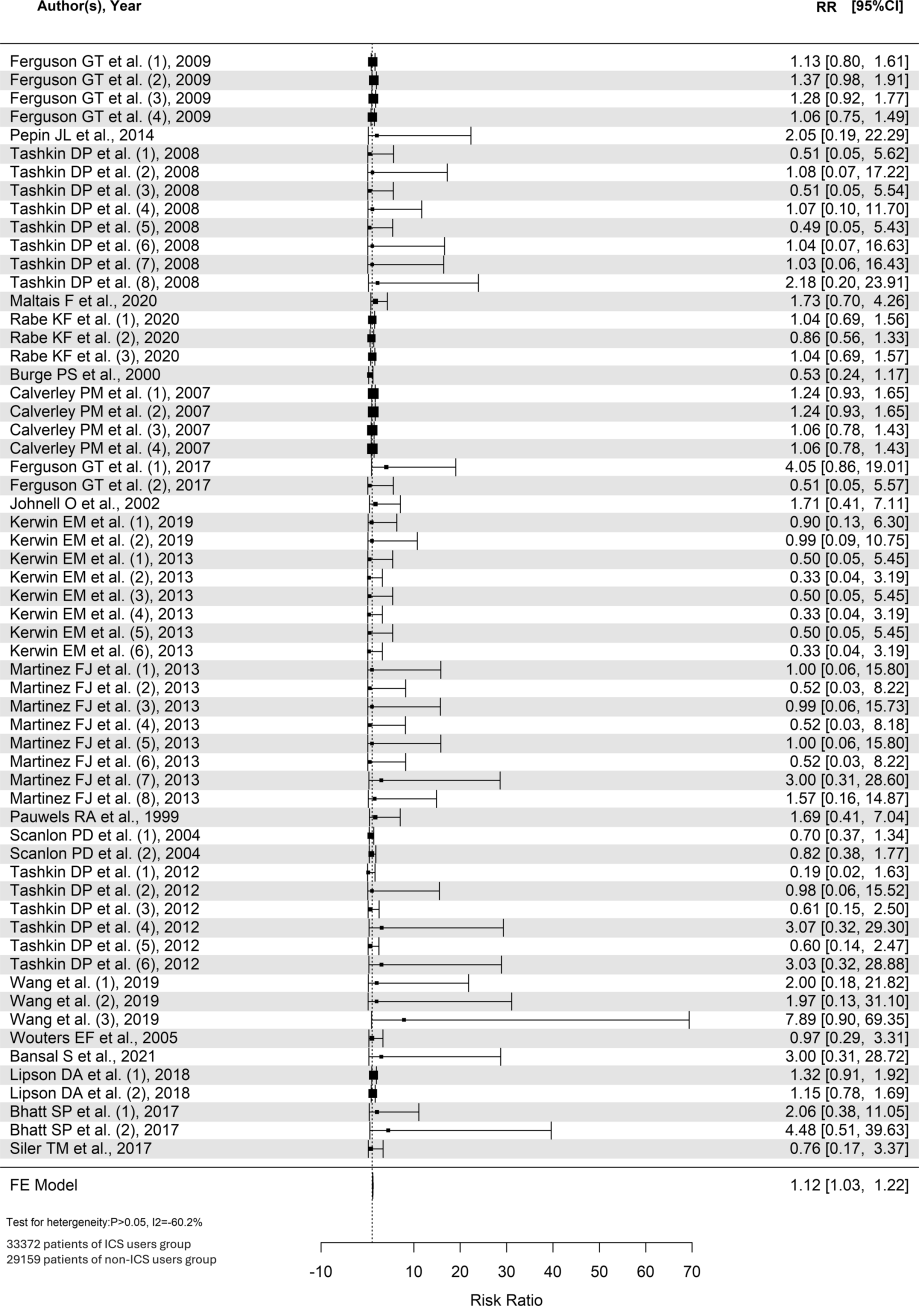
Forest plot for all doses of ICS in RCTs for osteoporosis or fracture.

In total, 21 randomized controlled trials (RCT) and 62 531 patients were included with osteoporosis or fracture as the outcome. Among all patients recruited in the RCTs, there were 33 372 patients included in the analysis of ICS‐exposed group, and 29 159 patients included in the analysis of ICS‐unexposed group. There were 3406 patients who developed osteoporosis or fracture in the ICS‐exposed group, and 865 patients developed osteoporosis or fracture in the ICS‐unexposed group within the RCTs. The RR for all ICS doses was 1.12 (95% CI = 1.03–1.22) (Figures 2 and S1). This indicated a significant increase of risk in osteoporosis or fractures under the exposure of ICS.

**FIGURE 3:**
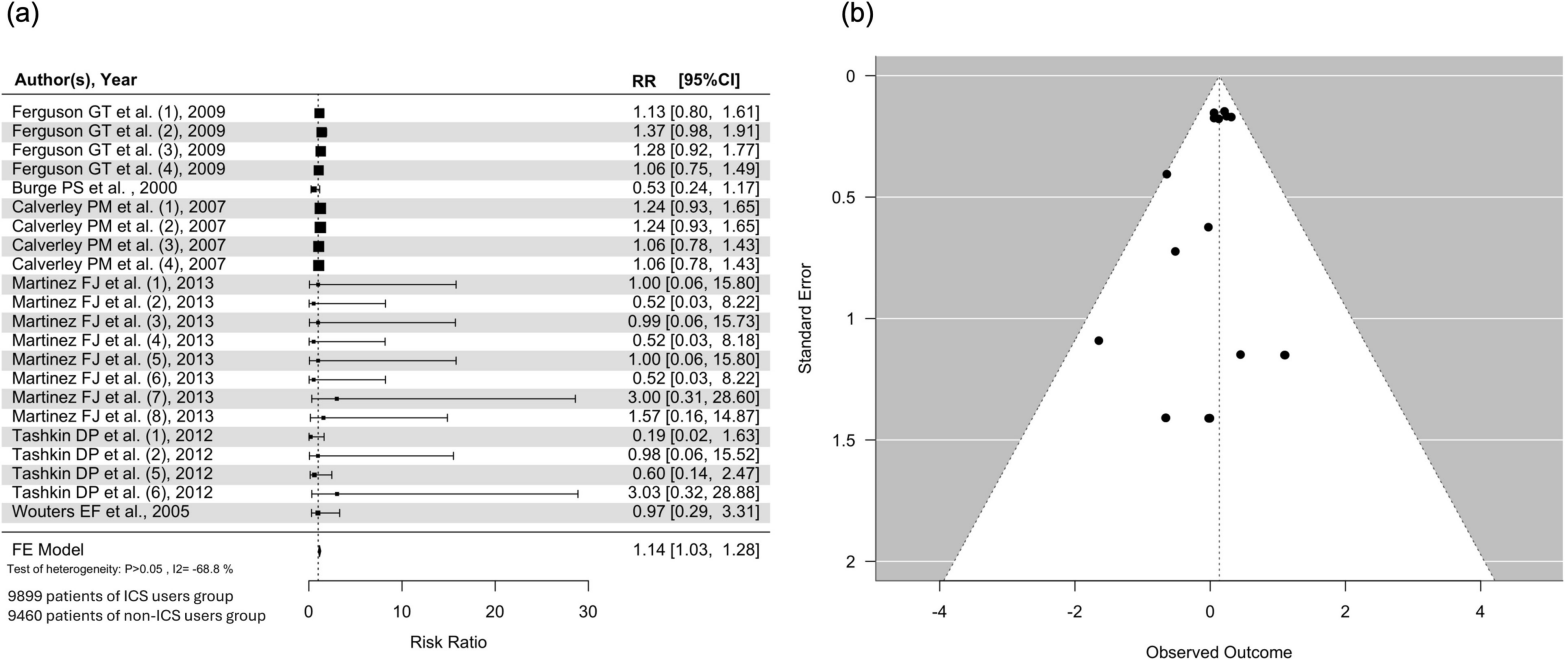
(a) Forest plot for high‐dose ICS in RCTs for osteoporosis or fracture, in RCTs reported fracture as outcome and in RCTs with moderate‐to‐severe COPD patients and (b) funnel plot for high‐dose ICS in RCTs for osteoporosis or fracture, in RCTs reported fracture as outcome and in RCTs with moderate to severe COPD patients.

In the subgroup of high‐dose ICS, there were 19 359 patients on high‐dose ICS, with 9899 in the ICS‐exposed group and 9460 in the ICS‐unexposed group. Among the ICS‐exposed group, 674 of them had fractures while none of them had osteoporosis; in the ICS‐unexposed group, 585 patients had fractures, but no osteoporosis cases were reported. The RR for high‐dose ICS was 1.14 (95% CI = 1.03–1.28) (Figure 3a,b), suggesting a significant increase of risk of fractures in the exposure of high‐dose ICS.

In the subgroup of medium‐dose ICS, there were 15 905 patients enrolled, with 7832 and 8073 patients in the ICS‐exposed and unexposed groups, respectively. Among the exposed group, there were 130 and 19 patients reported with fractures and osteoporosis cases, respectively. In the unexposed group, there were 123 and 20 cases of fractures and osteoporosis cases reported, respectively. The RR for medium‐dose ICS was 1.05 (95% CI = 0.83–1.31), suggesting an insignificant increase of risk in osteoporosis or fractures contributed by medium dose of ICS.

In the subgroup of low‐dose ICS, there were 22 470 patients on low‐dose ICS. Among them, 14 405 and 8065 patients were in the exposed and unexposed groups, respectively. A total of 2583 and 137 patients in the exposed and unexposed groups reported to have fractures, respectively, while none of them was reported to have osteoporosis. The RR for low‐dose ICS was 1.10 (95% CI = 0.90–1.35) (Figure S2). This suggested an insignificant increase of risk in fractures owing to the low‐dose ICS exposure.

The pooled estimates from the RCTs suggested a significant increase of risk in osteoporosis or fractures under ICS exposure. Particularly, only the high‐dose, but neither medium‐ nor low‐dose, ICS was significantly associated with increased risks for fracture in RCTs.

To separate the analysis for fracture risk, from the included RCTs, there were 19 RCTs reported fracture as the outcome. A total of 3387 patients who received ICS and 845 patients who did not receive ICS developed fracture within these RCTs. ICS was associated with higher risk for fracture, and the RR for developing fractures due to all ICS doses was 1.12 (95% CI = 1.03–1.23) (Figure S3a,b). Among the included RCTs studying high‐ and low‐dose ICS, there were no osteoporosis cases found and to be eliminated for analysis. While in the medium ICS dose group, after eliminating the osteoporosis cases for studying the fracture risk, the RR is 1.04 (95% CI = 0.81–1.33). In the subgroup analysis for the fracture development owing to different ICS exposures, it is found that there is a significant risk of having fractures due to all ICS exposures, RR = 1.12 (95% CI = 1.03–1.23). Of different dosages, high‐dose, but neither medium‐ nor low‐dose, ICS was significantly associated with increased risks for fracture in RCTs.

### 3.3 From Observational Studies

**FIGURE 4:**
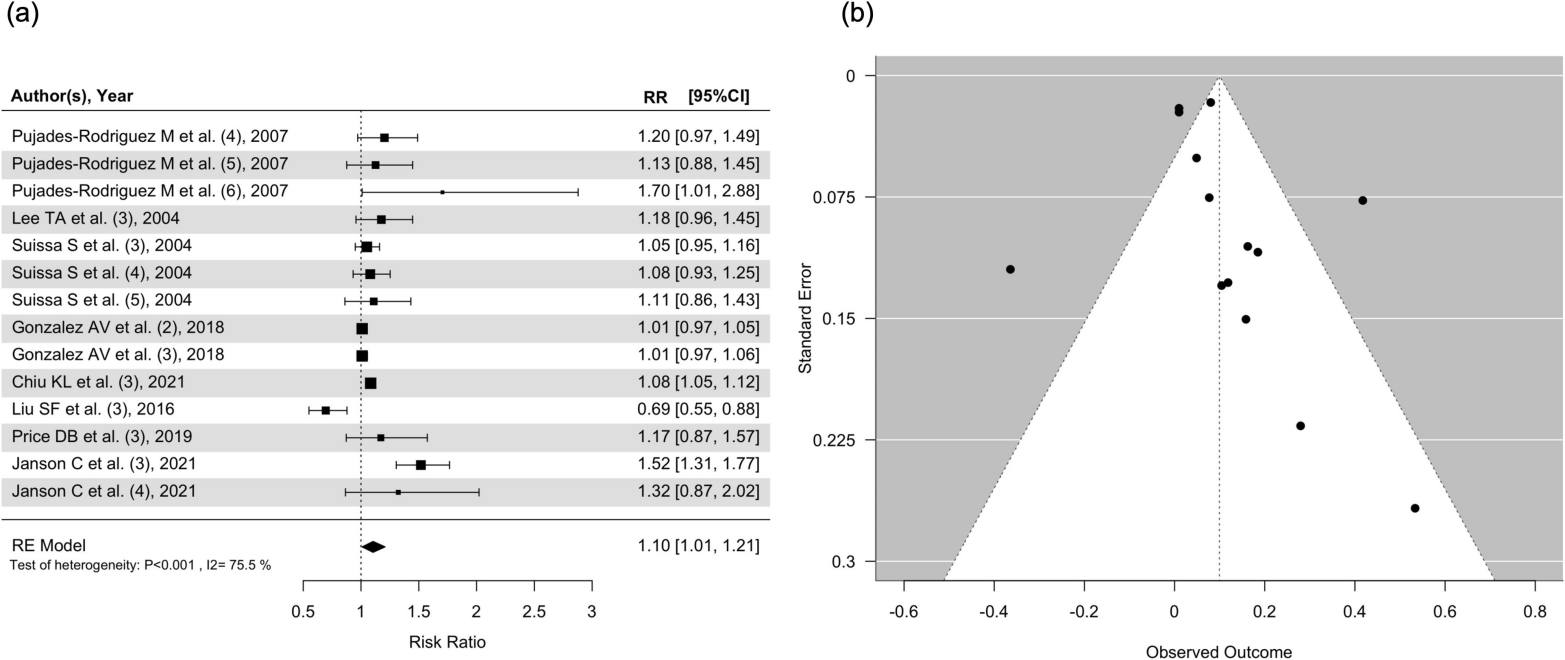
(a) Forest plot for high‐dose ICS in observational studies for osteoporosis or fracture and (b) funnel plot for high‐dose ICS in observational studies for osteoporosis or fracture.

Eight observational studies were included with osteoporosis or fracture as the outcome. From the included studies, 327 949 patients were counted to develop osteoporosis or fracture in the analysis. The RR for all ICS doses was 1.06 (95% CI = 0.98–1.14) (Figure 4a). Among the patients being included in the observational studies, 1 050 002 were on high‐dose ICS, with 101 903 of them reported to have osteoporosis or fracture. The RR for high‐dose ICS was 1.10 (95% CI = 1.01–1.21) (Figure 4a,b). A total of 558 436 patients were on medium‐dose ICS, with 72 879 patients having osteoporosis or fracture cases. The RR for medium‐dose ICS was 1.02 (95% CI = 0.884–1.174). There were 420 773 patients on low‐dose ICS, with 153 168 osteoporosis or fracture cases reported. The RR for low‐dose ICS was 1.02 (95% CI = 0.85–1.21) (Figure S4b). High‐dose, but neither medium‐ nor low‐dose, ICS was associated with significant increased risks for osteoporosis or fracture in observational studies without healthy controls.

In the subgroup of fractures cases, five observational studies had reported fracture as the outcome. There were 158 545 patients who developed fracture within these five observational studies. The RR for all ICS doses was 1.06 (95% CI = 1.00–1.12) (Figure S5a). Among the patients being included in the observational studies, 799 202 were on high‐dose ICS, with 41 321 of them having fracture. The RR for high‐dose ICS was 1.13 (95% CI = 1.03–1.24) (Figure S5b). A total of 333 930 patients were found on medium‐dose ICS treatment, and 18 532 of them reported to have fracture. The RR for medium‐dose ICS was 0.990 (95% CI = 0.951–1.029). There were 180 983 patients on low‐dose ICS, with 98 693 fractures. The RR for low‐dose ICS was 0.99 (95% CI = 0.90–1.09) (Figure S5c). High‐dose, but neither medium‐ nor low‐dose, ICS was associated with a significant increased risk for fracture in observational studies without healthy controls.

### 3.4 Subgroup Analysis

In order to assess the effect of prolonged use of ICS use and the associated risks of osteoporosis or fracture, a subgroup analysis was conducted on studies that included patients who had been on ICS for less than 1 year and at least 1 year. Eight RCTs were included in the subgroup analysis, and the RR for the group of patients who received all ICS doses for less than 1 year during the study period was 1.03 (95% CI = 0.67–1.68) (Figure S8), showing that less than 1‐year ICS use was not associated with an increased risk for osteoporosis or fractures. Also, 13 RCTs were included in the subgroup analysis, revealing that the RR for the group of patients who received all ICS doses for at least 1 year during the study period was 1.13 (95% CI = 1.03–1.23) (Figure S6). Apart from high‐dose ICS, receiving ICS for 1 year or longer was associated with an increased risk for osteoporosis or fractures.

Additionally, a subgroup analysis was performed in patients with moderate‐to‐severe COPD. Among the included RCTs and observational studies, 17 RCTs included patients with moderate‐to‐severe COPD. The RR for ICS use among patients with moderate‐to‐severe COPD and osteoporosis or fracture was 1.13 (95% CI = 1.03–1.23), *p*‐value < 0.01*) (Figure S7a,b). All patients were with moderate‐to‐severe COPD in the studies included for receiving high‐dose ICS. As a result, high‐dose ICS was found to be linked to osteoporosis or fracture in these patients, with an RR of 1.14 (95% CI = 1.03–1.28) and a *p*‐value = 0.015* (Figure 3a). Low‐dose ICS was not associated with osteoporosis or fracture among patients with moderate‐to‐severe COPD with RR of 1.08 (95% CI = 0.88–1.33) and a *p*‐value = 0.479 (Figure S7c).

## 4 Discussion

This systematic review and meta‐analysis of 21 RCTs and eight observational studies in patients with COPD demonstrated the possible dose‐related osteoporosis or fracture risks associated with ICS use. The findings highlighted the importance of appropriate ICS therapy in patients with COPD to prevent significant adverse events including osteoporosis or fracture.

The adverse events from ICS use in COPD are common and alarming. Yet, ICS remains an important pharmacotherapy in COPD especially in patients with eosinophilic phenotype with high exacerbation risks. As such, cautious and appropriate use of ICS, including choosing a safer one and using the lowest possible dose, should be considered. A recent systematic review and meta‐analysis suggested that high ICS dose neither reduces COPD exacerbation risk and mortality rates nor increases pneumonia risk relative to medium dosing [51]. It may not be appropriate to prescribe high‐dose ICS for patients with COPD. A lower dose may already be sufficient to prevent COPD exacerbation while it can also ameliorate the potential adverse effects such as osteoporosis or fracture.

We demonstrated the dose‐dependent risks of ICS on the development of osteoporosis or fracture in this systematic review and meta‐analysis. It clears up some of the concerns and controversies in the past. Corticosteroid exposure is well known to be associated with osteoporosis. However, systemic corticosteroid and ICS have a major difference in terms of systemic absorption. Our systematic review and meta‐analysis included RCTs and observational studies. They were separately analyzed with consistent results demonstrated. Only high‐dose ICS is reported to be associated with osteoporosis or fracture. Low‐dose ICS is considered to be safe with no increased risks of osteoporosis or fracture. For patients who are indicated for ICS use in COPD, low‐dose ICS would be a better option as it would be adequate to prevent COPD exacerbation while not increasing osteoporosis or fracture risks.

It is important to address the risks of osteoporosis or fracture among patients with COPD as they are important comorbidities in COPD. The potential contributing factors include smoking, reduced physical activity, low body weight and low Vitamin D level [52]. The development of fracture would lead to limitations in mobility, which will bring about negative impact in terms of physical activity and function in patients with COPD. It is crucial to screen for osteoporosis and prevent the development by removing all the possible secondary causes. While lifestyle modification will certainly help, the possible contribution from corticosteroid exposure cannot be forgotten. While the exposure to systemic corticosteroid for treating COPD exacerbation cannot be avoided, the sustained exposure to high‐dose ICS can certainly be avoided. Replacing high‐dose ICS by low‐dose ICS and appropriately withdraw ICS that is inappropriately prescribed shall be considered, given the possible adverse effects from high‐dose ICS use, with osteoporosis or fracture development.

One of the limitations of the meta‐analysis is that the duration of ICS treatment varies in the studies included. There were six studies that had ICS treatment and follow‐up to be less than 1 year, which was not associated with osteoporosis and fracture. Apart from the dose of ICS, the duration of ICS treatment is another factor that may influence the development of osteoporosis and fracture. Because of the study design of the included literatures, we could not examine the time from ICS use to the development of osteoporosis and fracture. This is an area that worth assessing as it can inform clinicians the association of ICS treatment duration and the development of osteoporosis and fracture and consider dose reduction for patients who have been treated for a long duration of high‐dose ICS, if appropriate.

## 5 Conclusion

High‐dose, but neither medium‐ nor low‐dose, ICS use in COPD is significantly associated with increased risks of osteoporosis or fracture.

## Author Contributions

Dr. Wang Chun Kwok and Ms Chung Ki Tsui was involved in the study concept and design, analysis and interpretation of data, acquisition of data, drafting of manuscript and approval of the final version of the manuscript. Dr. Isaac Sze Him Leung and Ms Shuk Man Ngai was involved in data analysis and interpretation, as well as the critical revision of the manuscript for important intellectual content. Dr. David Chi Leung Lam and Prof. Mary Sau Man Ip were involved in the critical revision of the manuscript for important intellectual content and approval of the final version of the manuscript. Dr. James Chung Man Ho was involved in the study concept and design, analysis and interpretation of data, drafting of manuscript, critical revision of the manuscript for important intellectual content, study supervision and approval of the final version of the manuscript.

## Disclosure

The authors have nothing to report.

## Ethics Statement

No ethical approval will be needed because data from previous published studies in which informed consent was obtained by primary investigators will be retrieved and analyzed.

## Conflicts of Interest

The authors declare no conflicts of interest.

## Name of Collaborators

The collaborators of this study are from the Department of Medicine, The University of Hong Kong, Queen Mary Hospital, 102 Pokfulam Road, Pokfulam, Hong Kong Special Administrative Region, China.

## Role of Sponsors

The authors have nothing to report.

## Other Contributions

The authors have nothing to report.

## Supporting information


**Figures S1** Funnel plot for all doses of ICS in RCTs for osteoporosis or fracture.

**Figure S2** Forest plot for low‐dose ICS in RCTs for osteoporosis or fracture, in RCTs reported fracture as outcome and in RCTs with moderate‐to‐severe COPD patients.

**Figure S3** (a) Forest plot for all doses of ICS in RCTs for fracture, in RCTs reported fracture as outcome and in RCTs with moderate‐to‐severe COPD patients and (b) funnel plot for all doses of ICS in RCTs for fracture, in RCTs reported fracture as outcome and in RCTs with moderate‐to‐severe COPD patients.

**Figure S4** (a) Forest plot for all doses of ICS in observational studies for osteoporosis or fracture and (b) forest plot for low‐dose ICS in observational studies for osteoporosis or fracture.

**Figure S5** (a) Forest plot for all doses of ICS in observational studies for fracture, (b) forest plot for high‐dose ICS in observational studies for fracture and (c) forest plot for low‐dose ICS in observational studies for fracture.

**Figure S6** Forest plot for all doses of ICS in RCTs for osteoporosis or fracture, in RCTs reported fracture as outcome and in RCTs with moderate‐to‐severe COPD patients; among subgroups received ICS for less than 1 year.

**Figure S7** Forest plot for all doses of ICS in RCTs for osteoporosis or fracture, in RCTs reported fracture as outcome and in RCTs with moderate‐to‐severe COPD patients; among subgroups received ICS for at least 1 year.

**Figure S8** (a) Forest plot for all doses of ICS in RCT and observational studies for fracture osteoporosis or fracture in subgroup of moderate‐to‐severe COPD patients, (b) funnel plot for all doses of ICS in RCT and observational studies for fracture osteoporosis or fracture in subgroup of moderate‐to‐severe COPD patients and (c) forest plot for low‐dose ICS in observational studies for fracture.

**Data S1** Supporting Information.

## Data Availability

Research data are not shared.
