# Supplementary figures and images for: Dose of Inhaled Corticosteroid in Chronic Obstructive Pulmonary Disease and Risks of Osteoporosis or Fracture—A Systematic Review and Meta‐Analysis
Source: Clin Respir J. 2025 May 26;19(5):e70086. doi: 10.1111/crj.70086 (PMC12106883; doi:10.1111/crj.70086)

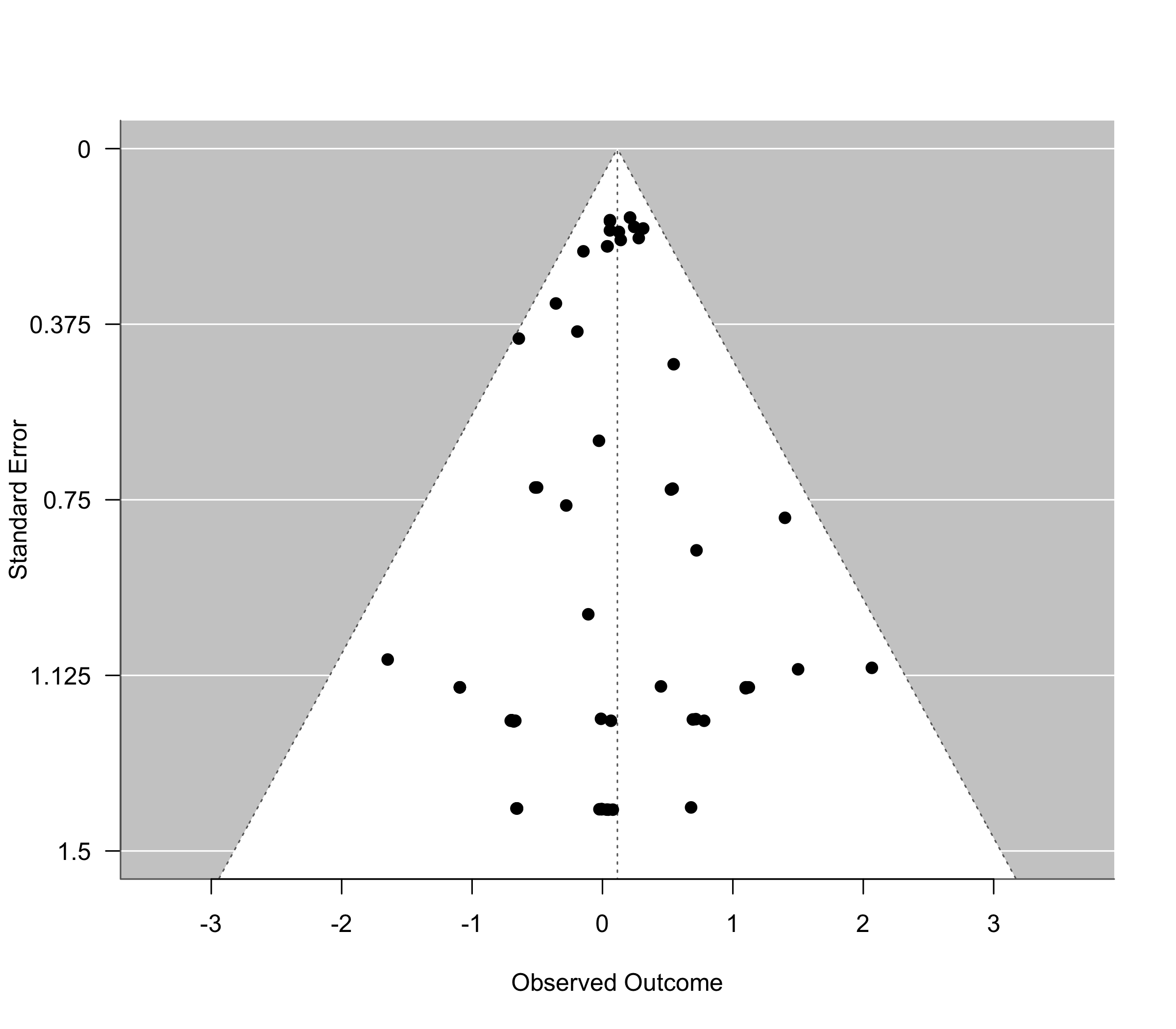

Supplement: Supplementary file 1 — Figures S1 Funnel plot for all doses of ICS in RCTs for osteoporosis or fracture. [file CRJ-19-e70086-s004.png]

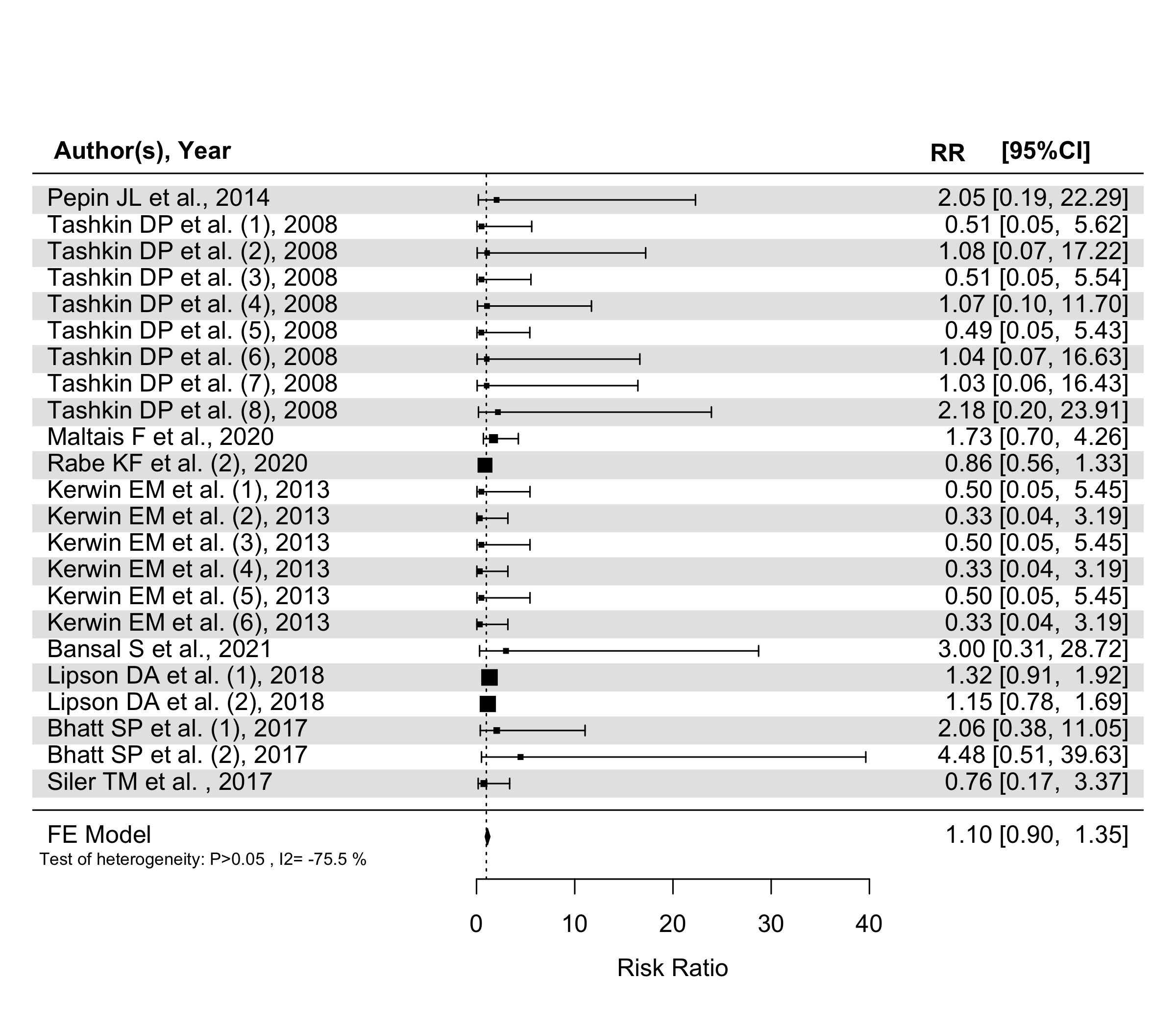

Supplement: Supplementary file 2 — Figure S2 Forest plot for low‐dose ICS in RCTs for osteoporosis or fracture, in RCTs reported fracture as outcome and in RCTs with moderate‐to‐severe COPD patients. [file CRJ-19-e70086-s006.png]

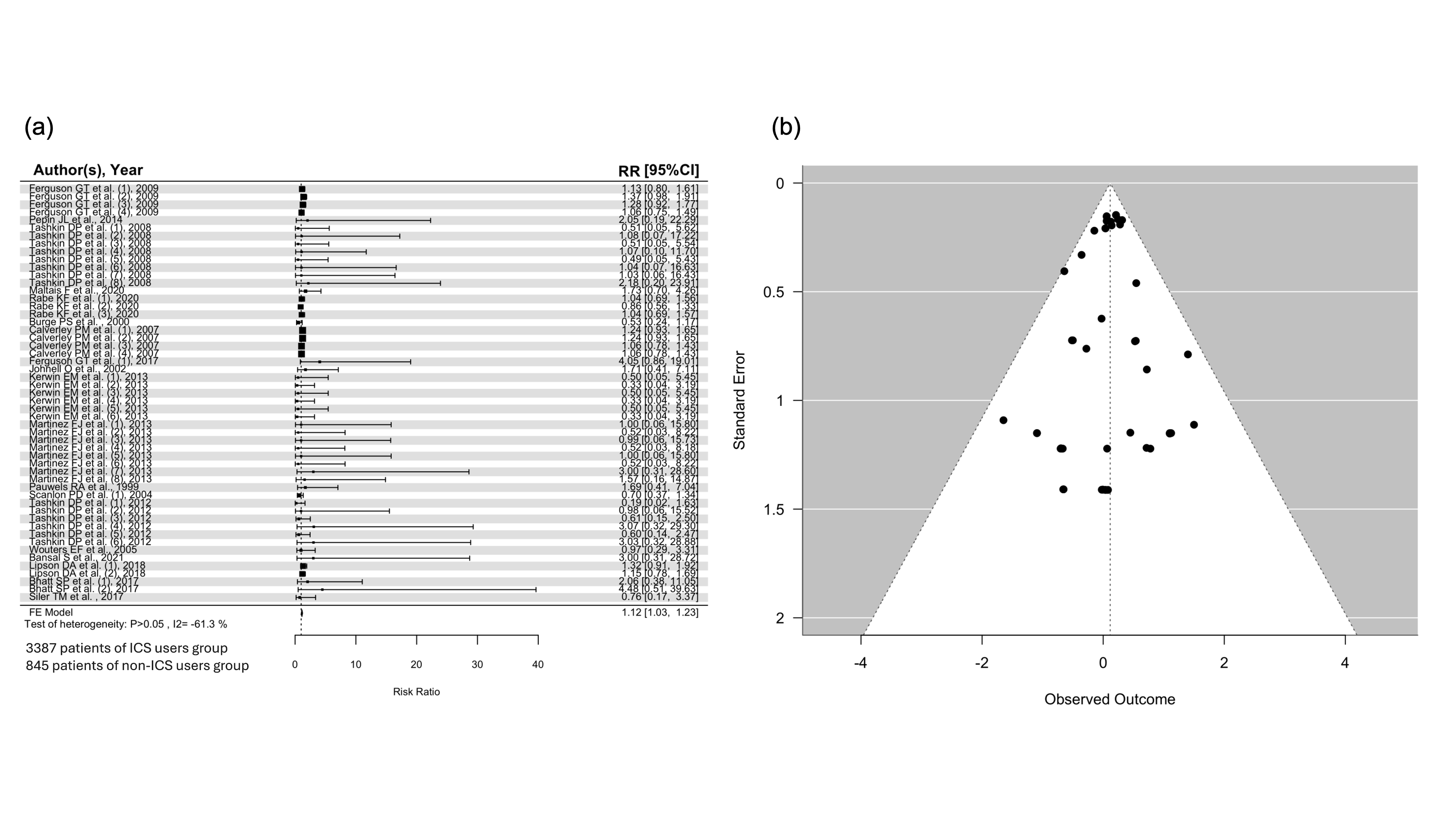

Supplement: Supplementary file 3 — Figure S3 (a) Forest plot for all doses of ICS in RCTs for fracture, in RCTs reported fracture as outcome and in RCTs with moderate‐to‐severe COPD patients and (b) funnel plot for all doses of ICS in RCTs for fracture, in RCTs reported fracture as outcome and in RCTs with moderate‐to‐severe COPD patients. [file CRJ-19-e70086-s009.png]

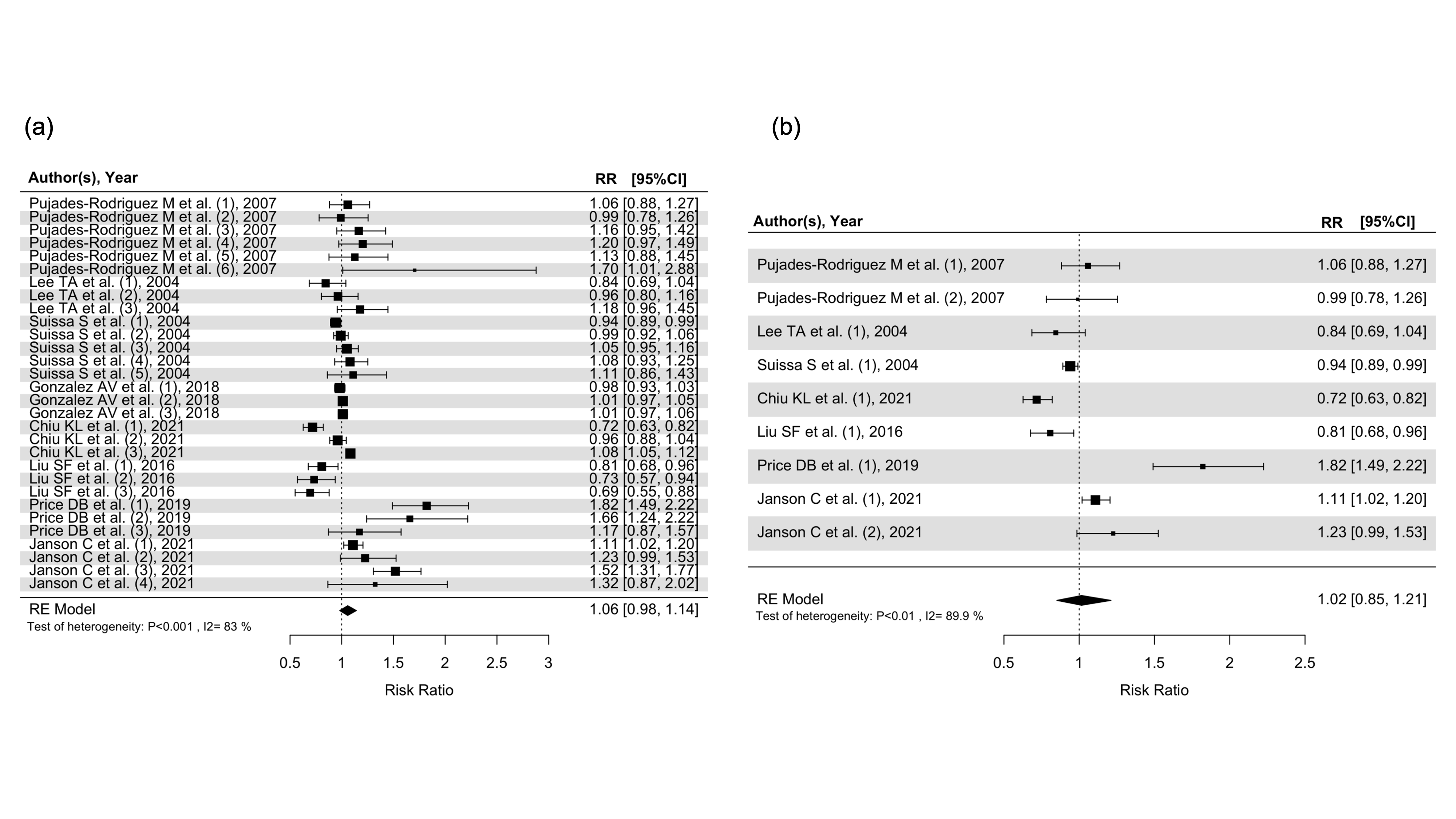

Supplement: Supplementary file 4 — Figure S4 (a) Forest plot for all doses of ICS in observational studies for osteoporosis or fracture and (b) forest plot for low‐dose ICS in observational studies for osteoporosis or fracture. [file CRJ-19-e70086-s001.png]

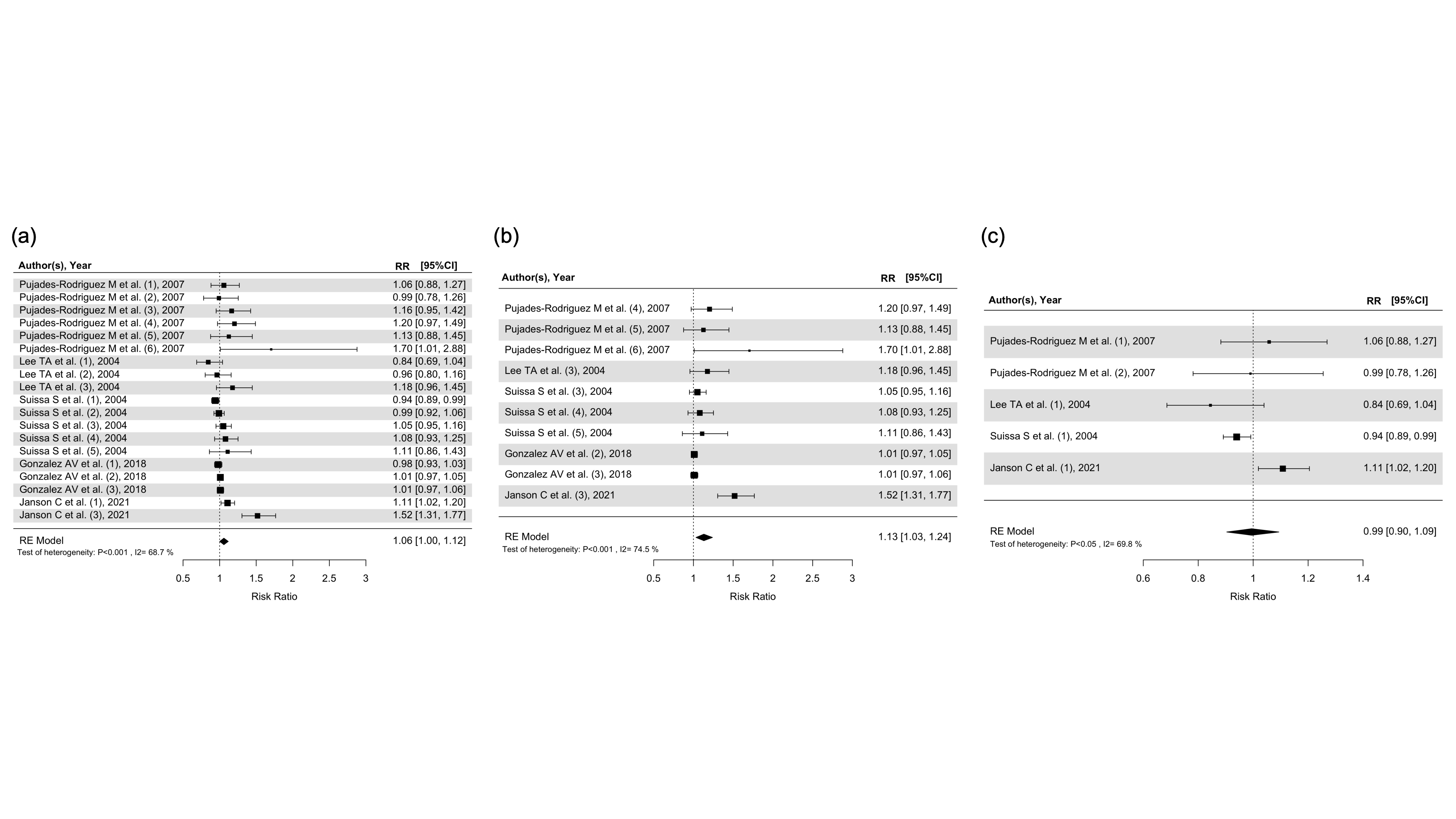

Supplement: Supplementary file 5 — Figure S5 (a) Forest plot for all doses of ICS in observational studies for fracture, (b) forest plot for high‐dose ICS in observational studies for fracture and (c) forest plot for low‐dose ICS in observational studies for fracture. [file CRJ-19-e70086-s003.png]

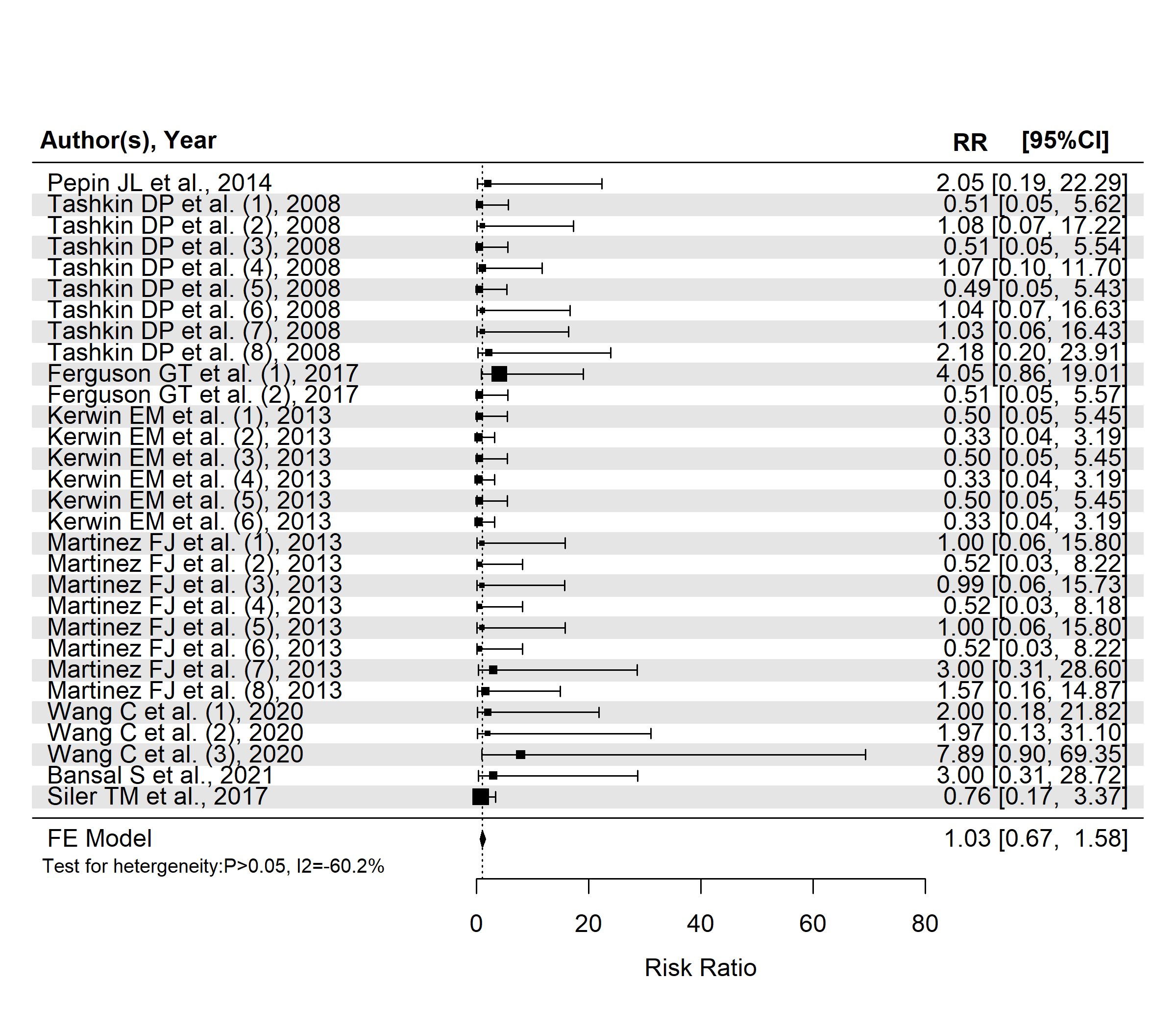

Supplement: Supplementary file 6 — Figure S6 Forest plot for all doses of ICS in RCTs for osteoporosis or fracture, in RCTs reported fracture as outcome and in RCTs with moderate‐to‐severe COPD patients; among subgroups received ICS for less than 1 year. [file CRJ-19-e70086-s008.png]

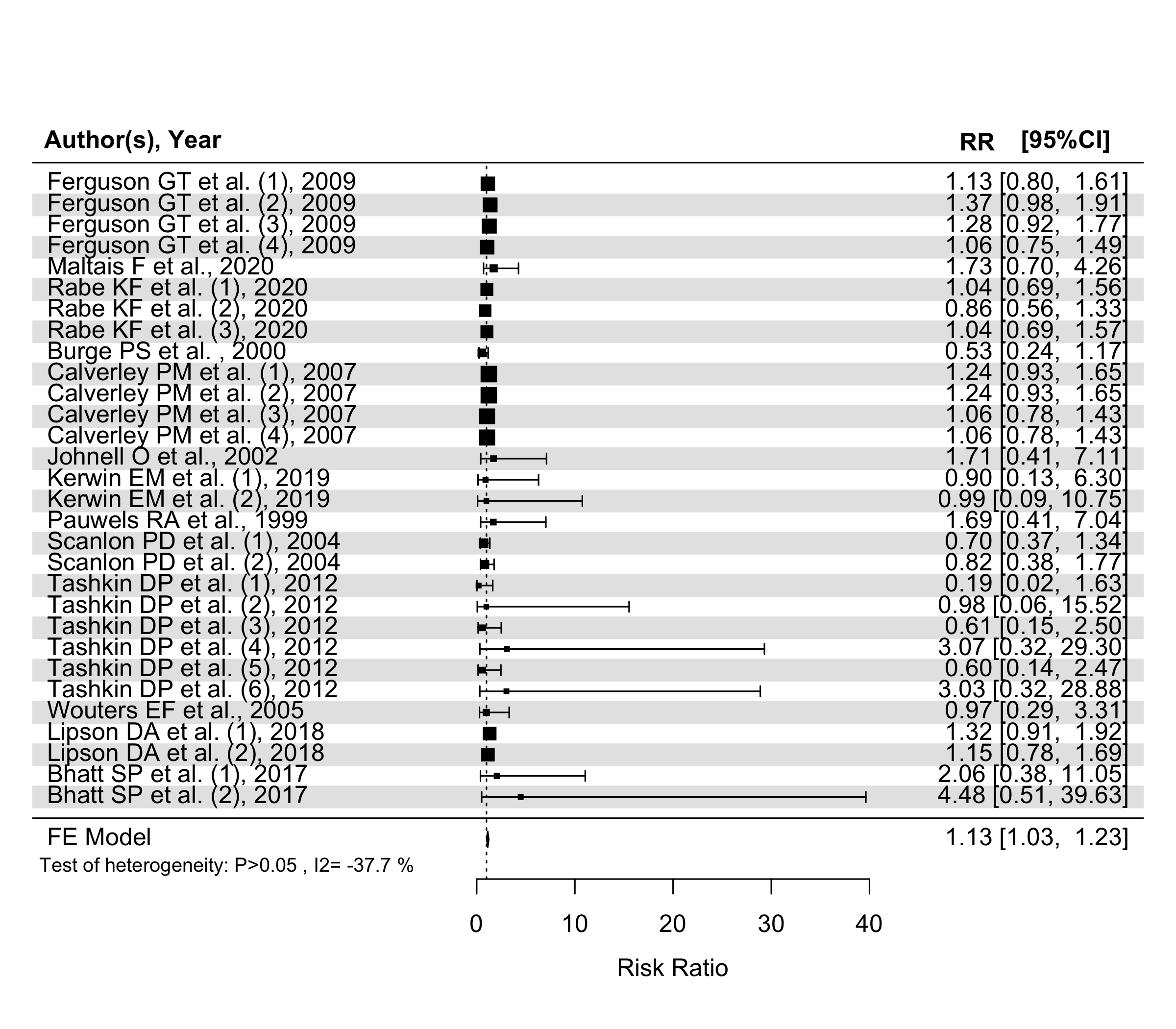

Supplement: Supplementary file 7 — Figure S7 Forest plot for all doses of ICS in RCTs for osteoporosis or fracture, in RCTs reported fracture as outcome and in RCTs with moderate‐to‐severe COPD patients; among subgroups received ICS for at least 1 year. [file CRJ-19-e70086-s007.png]

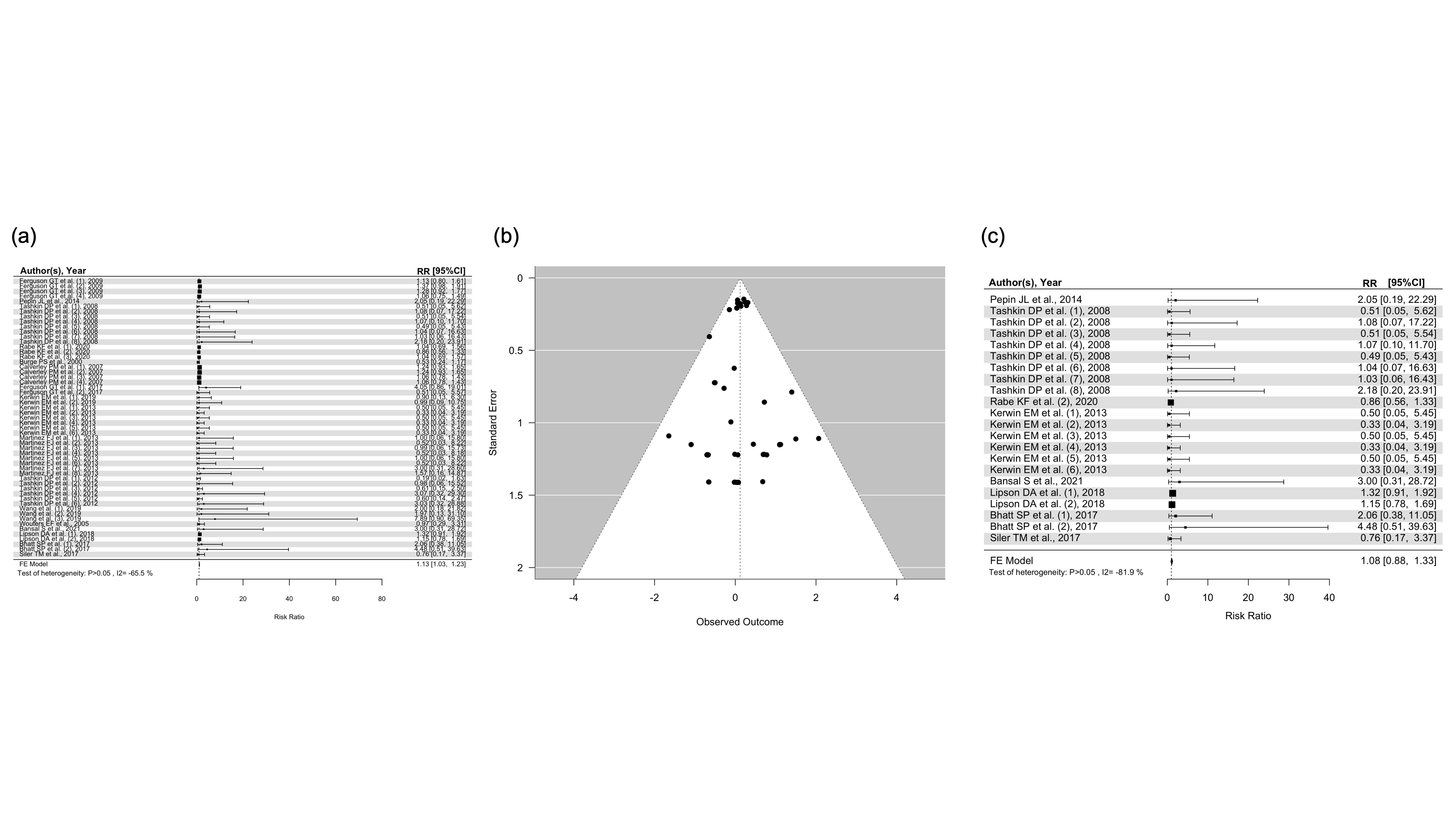

Supplement: Supplementary file 8 — Figure S8 (a) Forest plot for all doses of ICS in RCT and observational studies for fracture osteoporosis or fracture in subgroup of moderate‐to‐severe COPD patients, (b) funnel plot for all doses of ICS in RCT and observational studies for fracture osteoporosis or fracture in subgroup of moderate‐to‐severe COPD patients and (c) forest plot for low‐dose ICS in observational studies for fracture. [file CRJ-19-e70086-s005.png]
