# Supplementary material for: Dose of Inhaled Corticosteroid in Chronic Obstructive Pulmonary Disease and Risks of Osteoporosis or Fracture—A Systematic Review and Meta‐Analysis
Source: Clin Respir J. 2025 May 26;19(5):e70086. doi: 10.1111/crj.70086 (PMC12106883; doi:10.1111/crj.70086)
Supplement: Supplementary file 9 — Data S1 Supporting Information. [file CRJ-19-e70086-s002.docx]

Supplementary Figures

Supplementary Figures S1 Funnel Plot for all doses ICS in RCTs for osteoporosis or fracture

Supplementary Figure S2 Forest Plot for low dose ICS in RCTs for osteoporosis or fracture, in RCTs reported fracture as outcome, and in RCTs with moderate to severe COPD patients

Supplementary Figure S3 (a) Forest Plot for all dose ICS in RCTs for fracture, in RCTs reported fracture as outcome, and in RCTs with moderate to severe COPD patients (b) Funnel Plot for all dose ICS in RCTs for fracture, in RCTs reported fracture as outcome, and in RCTs with moderate to severe COPD patients

Supplementary Figure S4 (a) Forest Plot for all doses ICS in observational studies for osteoporosis or fracture (b) Forest Plot for low dose ICS in observational studies for osteoporosis or fracture

Supplementary Figure S5 (a) Forest Plot for all doses ICS in observational studies for fracture (b) Forest Plot for high dose ICS in observational studies for fracture (c) Forest Plot for low dose ICS in observational studies for fracture

Supplementary Figure S6 Forest Plot for all dose ICS in RCTs for osteoporosis or fracture, in RCTs reported fracture as outcome, and in RCTs with moderate to severe COPD patients; among subgroup received ICS for less than 1 year

Supplementary Figure S7 Forest Plot for all dose ICS in RCTs for osteoporosis or fracture, in RCTs reported fracture as outcome, and in RCTs with moderate to severe COPD patients; among subgroup received ICS for at least 1 year

Supplementary Figure S8 (a) Forest Plot for all doses ICS in RCT and observational studies for fracture osteoporosis or fracture in subgroup of moderate to severe COPD patients (b) Funnel Plot for all doses ICS in RCT and observational studies for fracture osteoporosis or fracture in subgroup of moderate to severe COPD patients (c) Forest Plot for low dose ICS in observational studies for fracture
